# A New Picture Process

**Published:** 1896-04

**Authors:** A. G. Field

**Affiliations:** Des Moines, Iowa


### A New Picture Process.

DES MOINES, IOWA, March 9, 1896.

*To the Editor:*—I have taken considerable interest in the published reports of the penetration of Roentgen's X rays and the application of the discovery to surgery, as well as to reports of similar results without the use of Crooke's tubes. By virtue of accident, I can report similar result independent of Crooke's tube, dynamo, or any other agent of electrical light or force, so far as I know. Some months ago I placed a piece of sensitized paper in a book and it was forgotten. When discovered a few days ago, the page of the book was clearly and legibly printed upon the sensitized paper, as a positive. The discovery was so bewildering that I did not think of fixing it at the time, but next morning, when I went to do so, the image had vanished, and I lost the evidence of what I saw. The experiment, however, can be repeated by any one. If the impression had been from ink or oil, it would not have faded.

A. G. FIELD, M.D.

In pulp exposure, place the devitalizing medicine in the dentine away from the point of exposure. You will thus secure devitalization with little pain, and very often more rapidly than if placed directly upon the exposed pulp. If the caries which exposes the pulp is upon the distal portion of a molar, then make a new cavity upon some mesial aspect of the tooth, and into that place the devitalizing medicine. This new cavity afterward gives direct entrance to anterior roots, which are usually necessary for a perfect operation.—*Western Dental Journal.*

